# Failure Detection in Quadcopter UAVs Using K-Means Clustering

**DOI:** 10.3390/s22166037

**Published:** 2022-08-12

**Authors:** James Cabahug, Hossein Eslamiat

**Affiliations:** School of Mechanical, Aerospace and Materials Engineering, Southern Illinois University Carbondale, 1230 Lincoln Dr, Carbondale, IL 62901, USA

**Keywords:** unmanned aerial vehicle, failure detection, faulty propeller, inertial measurement unit (IMU) sensor, vibration signal, k-means clustering, UAV safety

## Abstract

We propose an unmanned aerial vehicle (UAV) failure detection system as the first step of a three-step autonomous emergency landing safety framework for UAVs. We showed the effectiveness and feasibility of using vibration data with the k-means clustering algorithm in detecting mid-flight UAV failures for that purpose. Specifically, we measured vibration signals for different faulty propeller cases during several test flights, utilizing a custom-made hardware system. After we made the vibration graphs and extracted the data, we investigated to determine the combination of acceleration and gyroscope parameters that results in the best accuracy of failure detection in quadcopter UAVs. Our investigations show that considering the gyroscope parameter in the vertical direction (gZ) along with the accelerometer parameter in the same direction (aZ) results in the highest accuracy of failure detection for the purpose of emergency landing of faulty UAVs, while ensuring a quick detection and timely engagement of the safety framework. Based on the parameter set (gZ-aZ), we then created scatter plots and confusion matrices, and applied the k-means clustering algorithm to the vibration dataset to classify the data into three health state clusters—normal, faulty, and failure. We confirm the effectiveness of the proposed system with flight experiments, in which we were able to detect faults and failures utilizing the aforementioned clusters in real time.

## 1. Introduction

Cutting-edge advancements in control methods, processors, sensors and communications have enabled unmanned aerial vehicles (UAVs, or drones) to emerge as one the most promising technologies in recent years. With many applications ranging from crop spraying to industrial inspection, and from aerial videography to payload carrying, they are making many laborious tasks more efficient. However, regulators still prevent mass adoption of UAVs in public spaces because of the safety challenges; the main concern being if a drone suffers a mid-flight failure, it could crash and cause catastrophic damage to the people or properties nearby. We are designing safety measures to reduce the damage that UAVs can make when mid-flight failures happen. Further, these damages are not limited only to the environment, but also to the UAV itself and its payload. Failures can occur due to poor health and existing faults in the main components of a UAV [1]. Researchers address the challenge of reducing fatal UAV crashes by estimating their health status and diagnosing occurring faults using Predictive Maintenance and Prognostics and Health Management (PHM) in refs. [2,3]. Various learning algorithms with reliable performance also optimize the health of UAVs, such as the works in refs. [4,5,6,7].

To address the challenge of mid-flight system failures in UAVs, we propose a three-step safety framework that includes:

1. Detecting failures in the UAV;
2. Finding and selecting a safe landing zone in case a failure is detected in step 1;
3. Steering the vehicle to the selected landing zone in step 2, for landing.

In this paper, we specifically look at the first step, and propose a failure detection system that has an acceptable accuracy of detecting faults and is quick enough to detect failures such that the second and third steps are activated promptly and effectively. For this purpose, we want to accomplish the first step in less than 1 s. In other words, if the system detects a failure within one second, we consider that acceptable, and if it takes more than a second to detect a failure, that would be unacceptable. We identify any UAV failure by focusing on health monitoring and data acquisition of processed vibration signals to identify system changes that show failure or faults. The second step, finding an uncluttered and safe landing zone, involves the use of image processing algorithms such as YOLOv5 on videos and images from a camera, to select the best landing spot. Researchers recently published a study considering the second step in ref. [8]. In the third step, an independent propulsion system can steer the UAV to the uncluttered landing zone.

Fault and failure are sometimes used interchangeably in unmanned aerial vehicle literature; however, in this work, we distinguish them based on the following explanation. A fault is a defect in one of the system components that does not prevent the vehicle from continuing its operation. In contrast, a failure is a state where the system can no longer operate. A CNC rolling machine with a crack defect is an example of a fault, while a fractured helicopter driving gearbox is an example of a failure. UAVs that suffer any fatal crash have components that are subject to failure, which can happen for a variety of reasons. UAVs have three categories of failures: structural failures, electrical failures, and communication failures [9]. Each UAV consists of structural components that have mechanical functions, such as motors, propellers, and actuators. Motor failure can occur for any multi-rotor UAV if at least one motor malfunctions at some point during a flight [10]. Propeller failure occurs with damaged propeller pairs or with an unbalance of mass [11]. Actuator failure exists with a diagnosed fault for the actuator that cannot deflect control surfaces for fixed-wing UAVs [12]. Furthermore, electrical failures can also occur when the UAV has malfunctioning electrical components, such as sensors or batteries. Moreover, sensors that operate past their temperature or voltage range during a flight are prone to sensor failure [13]. Battery failure is also possible if the main UAV battery attains extremely poor quality with significant damage [14]. Moreover, communication failure includes issues from the GPS, radio, and controller components, where the controller loses communication despite the UAV being in range, when bad weather affects the UAV flight, or in areas where the signal is poor [15].

In this paper, we specifically look at quadcopter UAVs and cover the first step of the framework, UAV failure detection, to achieve the objective of increasing the safety and minimizing crashes in faulty UAVs. The goal of the safety framework is to minimize the damage to people and property, as well as the UAV and its payload. Here, we propose a fast and accurate failure detection system that uses an unsupervised learning algorithm, k-means clustering. Researchers implemented this algorithm in different UAV applications, such as horizon detection and fault diagnosis [16,17,18]. However, UAV failure detection with the k-means clustering algorithm is not seen in the literature, and our novel system is the first to address this algorithm for the safety framework of emergency landing for UAVs. To successfully detect UAV failure, we analyze vibration signals during a flight using an inertial measurement unit (IMU) sensor for detecting propeller failure. We consider different propeller fault configurations, as mechanical damage in the propellers causes large vibrations due to the presence of unbalanced forces. Then, we define three health states; normal, faulty and failure; we employ k-means clustering to classify the health of the UAV based on the acquired data. Our investigations show that this proposed method is efficient in finding failures quickly and accurately, while fulfilling the first-step of the safety framework. We also validate the UAV failure detection system in experimental flights using a light-emitting diode (LED) subsystem for a visual representation of the proposed algorithm.

The organization of the paper is as follows: Section 2 describes the related works and identifies the gap that we try to bridge; specifically using k-means clustering for fast and accurate failure detection as a part of an emergency landing safety framework for quadcopter UAVs. In Section 3, we thoroughly discuss the experimental setup for the experiments conducted. In Section 4, the proposed method for the UAV failure detection system is presented. In Section 5, results are shown and discussed, followed by the conclusion in Section 6.

## 2. Related Works

Different groups have performed research for UAV failure detection in experimental and simulated studies. For instance, the Air Lab Fault and Anomaly (ALFA) dataset identifies different failures, such as engine full power loss and control surface failures like rudder stuck to the left, for a fixed-wing UAV equipped with an onboard computer [19]. The first ground truth failure message for each control surface appears 0.2 s after the exact moment of the fault. A different group used horizontal takeoff and landing (HTOL) and vertical takeoff and landing (VTOL) UAVs for assessing failure in ref. [20], where a theoretical model analyzes a fixed-wing UAV spiraling down to the ground. Three prescribed circles for both HTOL and VTOL used safe path landing from the simulation. This study showed that crashes for HTOL UAVs have fewer fatalities than VTOL UAVs, since the calculated expected level of safety was higher for HTOL UAVs. A simulated flight controller used the reinforcement landing algorithm that trains a neural network model to detect mid-flight UAV failure for structural components with a recurrent neural network (RNN) and fault-tolerant bio-inspired flight controller (FT-BFC) [21,22]. Researchers plotted quadcopter position and speed for waypoint tracking; these algorithms helped detect failure within 2.5 s and achieved the desired waypoint in a shorter time. The method in ref. [23] also used a neural network model, but researchers took acoustic measurements of the UAV making noise, such as sound pressure level, in an anechoic chamber for extracting features. The model attained an accuracy of 0.9763 when detecting unbalanced UAV propeller blades.

Other researchers performed vibration analysis during the flight to monitor the health status of the UAV, where an accelerometer measured vibration signals, in the frequency domain, for different cases of defective propellers without the need for additional sensors [24]. This study considered single damaged and two broken propellers utilizing discrete Fourier transformation for measuring the signals; this method proved efficacious for identifying damaged propellers. In another study, signal processing detected the physical impairment of the Falcon V5 rotor blades using support vector machine (SVM) [25]. Their algorithm estimates the fault diagnosis of rotor blades model-free; of the three performance labels from the experimental verification, fast Fourier transform was the best in detecting failure for UAV propeller blades in 250 ms. In ref. [26], the IMU sensor also measured the in-flight vibration data of a fixed wing UAV for bearing faults to detect bearing failure. The study proved that the vibration anomaly indicator (VAT) is a useful diagnostic feature of UAV health monitoring, attaining positive values between 600 s and 1500 s. In ref. [27], a one-dimensional convolutional neural network (1D-CNN) deep learning algorithm identified the rotor fault of a UAV, which reconstructed a sample of vibration acceleration signals. The method that used 1D-CNN algorithms was more efficient than traditional signal analysis and modeling methods, as the algorithm achieves accuracies as high as 86%. In ref. [4], researchers detected failure using the self-organizing map (SOM) algorithm which measures its health status using acceleration and gyro sensors for the propeller and motor states. Clustered graphs contained chosen neurons for failure classifications, and the SOM model had an accuracy of 99% and recall of failure situation of 100% in this study. In ref. [7], fault detection and identification techniques for quadcopters utilized airborne acceleration sensors for measuring airframe vibration signals, and long- and short-term memory (LSTM) and back propagation (BP) algorithms used test and training samples to detect propeller faults. Between the two models, the LSTM model was more accurate than the BP model, since the accuracy of the LSTM model was 96%, and the BP model was 65%.

In ref. [28], a hall sensor measures current from brushless direct current (BLDC) motors for detecting a small inter-turn fault in a quadcopter. The skewness scanning algorithm for multi-resolution analysis determines the number of shorted-turns as roll, pitch, and yaw, and inputs monitor skewness values. Researchers selected the skewness of approximate coefficients for levels 5 and 6 (SA5 and SA6) for pitch and levels 5, 6, and 8 (SA5, SA6, and SA8) for yaw for detecting the number of shorted-turns in the BLDC motor since their skewness values were precise. In refs. [29,30], researchers measured the density of vibration peaks for signal analysis based on chaos using density of maxima (SAC-DM) using an onboard accelerometer to estimate the BLDC motor speed. They measured SAC-DM values and achieved failure detection in one second, as the average accuracy attained was 82.75%. In ref. [31], researchers implemented a dynamic fault detection algorithm for partial and complete motor failure. Their quadrotor with enabled fault tolerant control (FTC) achieved smooth landing as the drone regulated the attitude in π/6 radians. In other words, the measured roll and pitch angles were between −π/6 and π/6 radians for FTC analysis. If motor failure exists, then a redundant flight recovery system (RFRS) monitors the motor health and tries to complete UAV operation [32]. As the number of working rotors changed from eight to four, RFRS was active in providing precise pitch and roll angles for *X*_odd_ (motors 1, 3, 5, and 7) and *X*_even_ (motors 2, 4, 6, and 8) flight attitudes. The measured vibration signal of a UAV motor improves the performance and stability of a UAV, as researchers made a correlation between stability and motor vibration [33]. The vibration amplitude of the faulty motor was 0.169 mm/s^2^, compared to the qualified motor of 0.0746 mm/s^2^, which shows that higher vibration amplitudes indicate UAV failure. Furthermore, the Arduino microcontroller obtained data for motor current signals and propeller vibration signals from current sensors and accelerometer based on different fault configurations [34]. The Q-learning and genetic algorithm (QFAM-GA) had the best performance of 94%, compared to the other algorithms in the study.

Table 1 outlines the relevant sources that compare the UAV used, the different types of failure, and the algorithm implemented. Based on simulated and experimental studies from the literature review, we found that structural failure, especially propeller and motor failure, is the most common type of failure. These studies use distinct configurations for the number of working propellers and motors. In general, UAVs that suffer a fatal crash typically have a failure in structural components. Supervised, unsupervised, and reinforcement learning algorithms create different classifications for the datasets. The combination of different algorithms, datasets, and extracted features ensues different results. In our study, we use a quadcopter to detect propeller failure, and our algorithm uses k-means clustering. Although this algorithm is simple and versatile, it arranges datasets in their respective clusters to determine the degree of existing UAV faults. We propose to include a visual representation of the working algorithm with an LED subsystem that contains three LEDs. We specify boundary conditions for a scatter plot that uses k-means clustering where one LED turns on at a time. To our knowledge, our study is the first one to use k-means clustering and an LED subsystem for UAV failure detection.

Although researchers achieved accurate results from using working algorithms and state of the art methods, the literature also mentions limitations of UAV failure detection methods. In ref. [21], recovery for propeller failure was harder to accomplish because of insufficient thrust and lost degree of freedom. In ref. [22], the quadcopter interceded sudden changes in position when changing the number of working motors, which caused greater noise. A modulated broadband noise between the 2 and 20 kHz frequencies had the highest noise values, which occurred due to unstable aerodynamics for the interaction of the air flow with the drone frame [23]. The fault detection system was not capable of determining the exact rotor that failed since the center of the UAV had the IMU sensor [25]. In ref. [27], the final recognition effect for weaker fault categories was insufficient in using a sequential sampling method. In ref. [29], the SAC-DM method had difficulty in achieving failure diagnosis.

## 3. Experimental Setup

### 3.1. Hardware System Design

**Figure 1:**
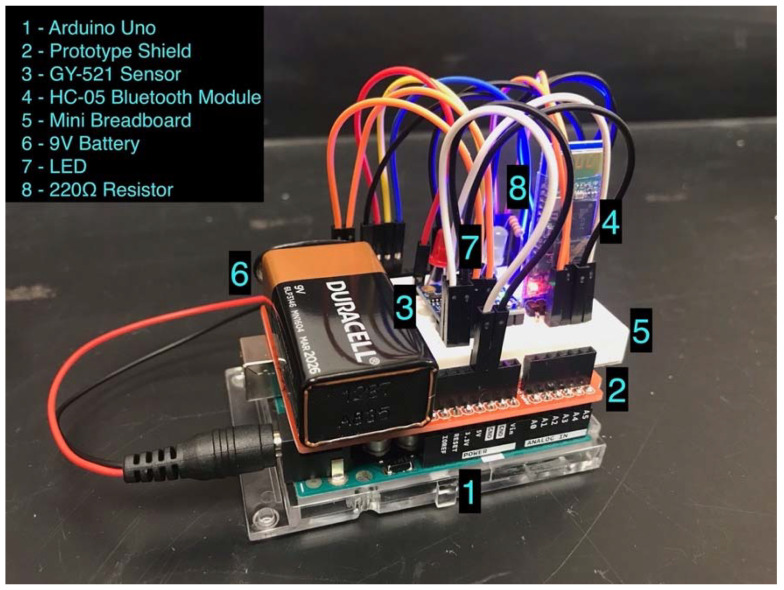
Designed Hardware System.

We mounted a custom-made hardware system onto a quadcopter to successfully measure vibration and detect failure. For each conducted experiment, we used Parrot ANAFI as the quadcopter, which is foldable, lightweight, and easily deployable. Figure 1 shows the hardware system with the following labeled parts: Arduino Uno, prototype shield, GY-521 sensor, HC-05 Bluetooth module, mini breadboard, Duracell 9 V battery, LED, and 220 Ω resistor. Square fasteners placed the hardware system onto the Parrot quadcopter before the flight experimentation began.

Arduino Uno Rev3 was the microcontroller board with an ATmega328P processor that uses data acquisition. It had 14 digital input/output pins and 6 analog pins for electronic connections. The corresponding software for this microcontroller board is Arduino IDE, which is capable of reading sensor data through a written code. The prototype shield was a base that housed the mini breadboard and 9 V battery.

Moreover, the GY-521 sensor included a 3-axis accelerometer, a 3-axis gyroscope, a digital motion processor, and a temperature sensor, which collected the vibration signals from the quadcopter. Variables aX, aY, and aZ represent acceleration in the *x*-, *y*-, and *z*-directions, and variables gX, gY, and gZ represent gyroscope data in the *x*-, *y*-, and *z*-directions, respectively. We soldered the GY-521 sensor to a pin header, which was placed on the mini breadboard. The HC-05 Bluetooth module transferred the vibration data from the GY-521 sensor to Arduino IDE, with available Bluetooth on the computer. The mini breadboard contained three LEDs (one blue LED, one yellow LED, and one red LED) which detected different health states for the LED subsystem (Section 4.4 provides a thorough explanation of the LED subsystem). Three 220 Ω resistors regulated the flowing current for each LED. Although a USB cable supplied power to the Arduino Uno, the Duracell 9 V battery was the power source we used, which helped provide power to the GY-521 sensor, the HC-05 Bluetooth module, and the LEDs during the flight.

**Figure 2:**
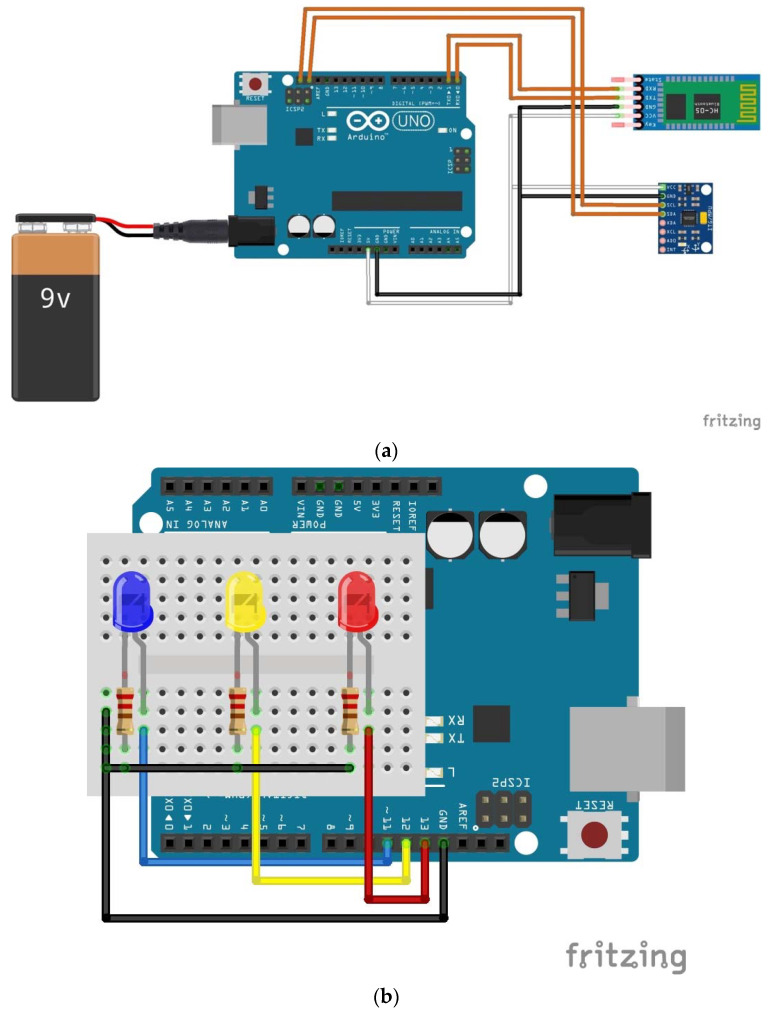
Schematic of Hardware Setup for (**a**) Sensor and (**b**) LEDs.

Figure 2 shows the electrical schematics for the labeled components.

Jumper wires, 10 cm in length, made the following pin connections. In Figure 2a, for the GY-521 sensor connecting to Arduino Uno, VCC goes to 5 V, GND goes to GND, SCL goes to SCL, and SDA goes to SDA. For the HC-05 Bluetooth module, VCC goes to 5 V, GND goes to GND, RXD goes to pin 0, and TXD goes to pin 1. In Figure 2b, the positive ends of the blue, yellow, and red LEDs connected pins 11, 12, and 13 on Arduino Uno, respectively. The 220 Ω resistors connected the negative ends of each LED and GND.

### 3.2. Cases for Propeller Faults

**Figure 3:**
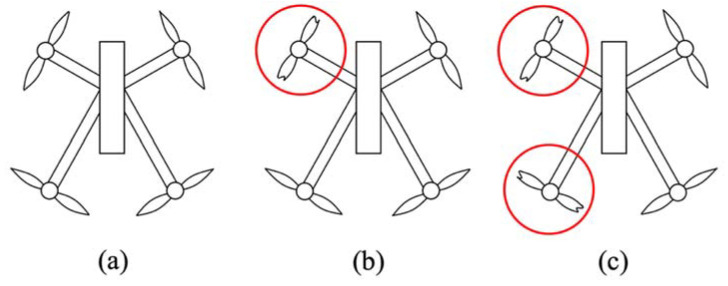
Cases for Propeller Faults with (**a**) Healthy, (**b**) One Set of Faulty Propellers (1SFP), and (**c**) Two Sets of Faulty Propellers (2SFP).

We flew the Parrot ANAFI quadcopter to collect vibration data needed for failure detection that prevents unintended crashing in urban and rural environments. We proposed different cases of propeller faults with 7 mm cut propellers. Faulty propellers replaced normal propellers using a small wrench in between flights. This study considers three propeller fault cases for the main experiment of measuring vibration: zero sets of faulty propellers (healthy), one set of faulty propellers (1SFP), and two sets of faulty propellers (2SFP) (see Figure 3).

For the first case (healthy), the quadcopter flew smoothly, establishing all working propeller sets. We expected minor faults and zero failure to occur when conducting these flight experiments. For the second case (1SFP), one rotor had two propellers with 7 mm cuts on both ends. The faulty propellers affected the total thrust and the overall dynamics for the Parrot ANAFI quadcopter. We expected moderate faults and possible failure to occur as the IMU sensor measures higher vibration magnitudes than the previous case. For the third case (2SFP), we attached two faulty propellers to two rotors. We expected severe faults and obvious failure to occur, due to the sensor measuring higher vibration magnitudes.

## 4. UAV Failure Detection System

**Figure 4:**
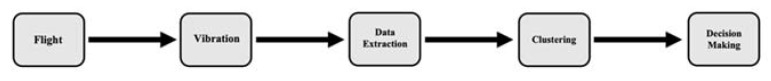
Block Diagram for Failure Detection Model.

In this paper, we developed a novel UAV failure detection system as a part of the safety framework to address safety concerns for communities and reduce the number of catastrophic UAV crashes. We propose the following five steps to successfully detect failure (see the block diagram in Figure 4):

Step 1: Conduct Flight Operations (Flight)
Step 2: Perform Vibration Measurements (Vibration)
Step 3: Extract Data using Arduino (Data Extraction)
Step 4: Apply Clustering Algorithm (Clustering)
Step 5: Decision-Making for Failure Detection (Decision-Making).

**Figure 5:**
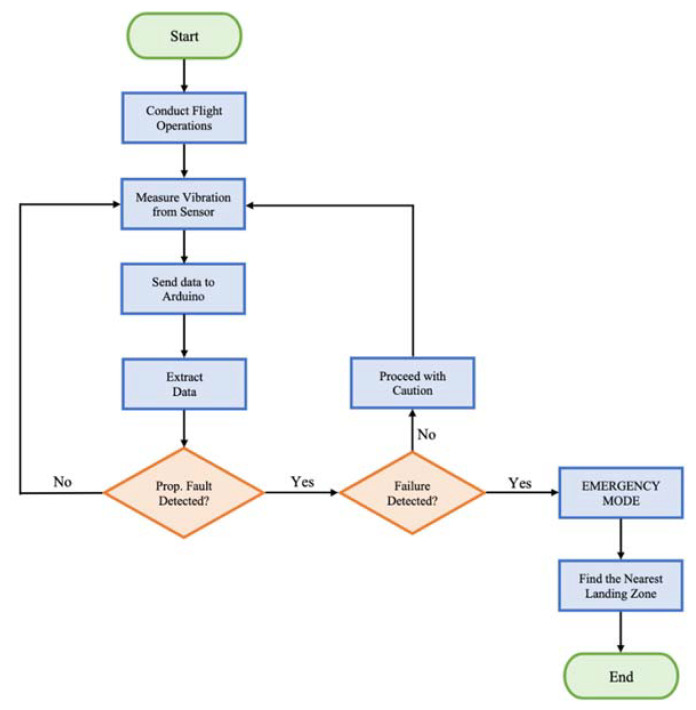
UAV Failure Detection System Flowchart.

Figure 5 shows the UAV failure detection model flowchart for utilizing the five steps. The vibration measuring process involves the GY-521 sensor measuring in-flight vibration, sending the vibration data to Arduino, and data extraction. The first orange diamond block looks at propeller fault detection, and the second orange diamond looks at failure detection.

If none of the propellers have any fault, then the vibration measuring process occurs again in several iterations until the flight is over. Otherwise, if propeller fault exists without failure, the quadcopter would proceed with caution. In the case that the system detects both propeller fault and failure, the quadcopter engages in emergency mode, and the nearest safe landing zone is found immediately to avoid catastrophic crashes.

### 4.1. Conduct Flight Operations

The Parrot ANAFI quadcopter must fly to detect failure and reduce the number of catastrophic crashes in urban and rural environments. We flew the quadcopter inside a large, enclosed cage in an indoor lab, and we performed experiments with different propeller cases. The quadcopter had 15 flights, where the healthy, 1SFP, and 2SFP propeller fault cases had five flights each, and a flight time of 4 min each. Given that we used the 9 V battery and attain 0.14 A for the measured current, the power that the failure detection system used was 1.26 W. Before flying the quadcopter, we secured the hardware system onto the drone, and we placed the quadcopter in the center of the enclosed cage. We recharged the drone when it approached low battery in between flights. We did not use the LEDs in this step, but we used them in the final step, decision-making for failure detection.

### 4.2. Perform Vibration Measurements

**Figure 6:**
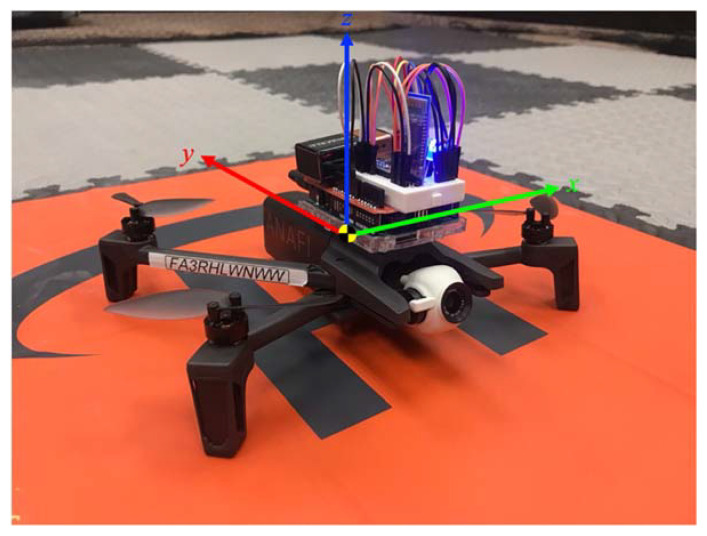
Quadcopter and Hardware System Coordinate System.

Figure 6 shows the Cartesian coordinate system for the quadcopter and hardware system combined, where the coordinate system directs the *x*- and *y*-axes along the horizontal plane and directs the *z*-axis orthogonal to the horizontal plane. The GY-521 sensor from the hardware system measures in-flight vibration signals for acceleration and gyro in the *x*-, *y*-, and *z*-directions. Next, the HC-05 Bluetooth module sends the vibration data from the Arduino Uno microcontroller to the computer, and Arduino IDE reads the vibration data, which contains three different propeller fault configurations (healthy, 1SFP, and 2SFP).

### 4.3. Extract Data Using Arduino

**Figure 7:**
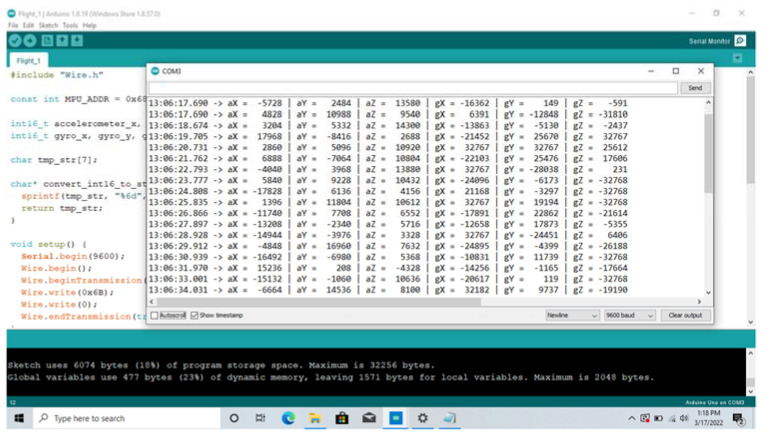
Running the Program in Arduino.

Arduino IDE displays the following six parameters with a written C++ code during each flight: aX, aY, aZ, gX, gY, and gZ (see Figure 7). We extracted the data by copying and pasting each parameter value into Excel. The input for each vibration graph is timestep (in minutes), and the output is one of the measured parameters (in least significant bit, LSB). Each vibration graph shows recorded data from the three propeller cases (healthy, 1SFP, and 2SFP). Since we obtained 15 flights in total during the experiment, we recorded data for five sets of three flights (S1 to S5).

### 4.4. Apply Clustering Algorithm

We performed data analysis for each vibration graph with the k-means clustering algorithm to classify different health states into *k* clusters given *n* objects. We aimed to minimize the Euclidean distance between each data point and the nearest centroid. Let $x_{i}$ be the training set, $\mu_{j}$ be the centroid set, and *J* be the objective function.

(1) $$J=\sum_{j=1}^{k}\sum_{i=1}^{m}\|x_{i}^{\left(j\right)}-\mu_{j}{}^{2}\|$$

Additionally, let $c^{\left(i\right)}$ be the output cluster vector. Minimizing the objective function finds the centroid value.

(2) $$c^{\left(i\right)}=arg_{j}min\;\|x_{i}^{\left(j\right)}-\mu_{j}{}^{2}\|$$

(3) $$\frac{\partial J}{\partial \mu_{j}}=-2\sum_{i=1}^{m}\left(x_{i}^{\left(j\right)}-\mu_{j}\right)=0$$

(4) $$\mu_{j}=\frac{\sum_{i=1}^{m}x_{i}^{\left(j\right)}}{\sum_{i=1}^{m}\left(1\right)}$$

In ref. [35], the authors fully explain this algorithm. We investigated different parameter combinations to evaluate the best parameter set for failure detection in faulty UAVs as the first step of the three-step safety framework for faulty UAVs, and applied the k-means clustering algorithm to this chosen parameter set. We also investigated whether the algorithm was fast and accurate enough for the first step of the safety framework and confirmed the effectiveness and feasibility of the proposed system for use in the safety framework by conducting experimental flights.

We used the dataset for k-means clustering involving parameter sets that have a selection of six measured parameters. Let *n* be the number of parameters selected, or dimension, where $n\in \left\{1,2,3,4,5,6\right\}$. Each dataset would have 750 data points from the fifteen flights, written as $\left(x_{1},\ldots ,x_{n}\right)$. For instance, if the dataset contained the parameters gY, gZ, and aZ, we would write the data points as $\left(x_{1},x_{2},x_{3}\right)$, where *x*_1_ is the gY value, *x*_2_ is the gZ value, and *x*_3_ is the aZ value. Excel presented the datasets as we carefully selected 50 output values from each vibration curve of the selected parameters, writing it as $\left(x_{1},\ldots ,x_{n}\right)$. We took the absolute value for all the data points prior to using the k-means clustering algorithm. Table 2 shows different combinations of parameter sets for this paper.

We imported each dataset into MATLAB, and we wrote a code for applying the k-means clustering algorithm with *k* = 3 and *n* = 750 to the datasets. Scatter plots titled “Quadcopter Failure Detection Clusters” (QFDC) showed three clusters: normal state, faulty state, and failure state. For computational complexity for the k-means clustering algorithm with *d* = 2 attributes, the time complexity is *O*(4500), and the space complexity is *O*(753).

For each QFDC plot, we used 3 × 3 confusion matrices for the failure detection system (see Table 3), where the letters F, A, and N denote “failure state,” “faulty state,” and “normal state” for the three health states, respectively. Data points that have the health state classified before importing in MATLAB have one “actual label.” In contrast, data points grouped into different clusters after running the MATLAB code with k-means clustering have one “clustering label.” Both the actual label and the clustering label contain the three health states. The leftmost column in Table 3 is the actual label, which uses the first letter in each cell, and the topmost row is the clustering label, which uses the second letter in each cell. Each data point compares the health state between the actual label and the clustering label for the confusion matrix. For instance, “AF” means that the actual label is the fault state, while the clustering label is the failure state. Equations (5)–(8) show performance metrics for recall, precision, accuracy, and F-Score, given the 3 × 3 confusion matrix.

(5) $$\mathrm{Recall}=\frac{\mathrm{FF}}{\mathrm{FF}+\mathrm{AF}+\mathrm{NF}}$$

(6) $$\mathrm{Precision}=\frac{\mathrm{FF}}{\mathrm{FF}+\mathrm{FA}+\mathrm{FN}}$$

(7) $$\mathrm{Accuracy}=\frac{\mathrm{FF}+\mathrm{AA}+\mathrm{NN}}{\mathrm{total}}$$

(8) $$F-\mathrm{Score}=\frac{2*\mathrm{Recall}*\mathrm{Precision}}{\mathrm{Recall}+\mathrm{Precision}}$$

### 4.5. Decision-Making for Failure Detection

**Figure 8:**
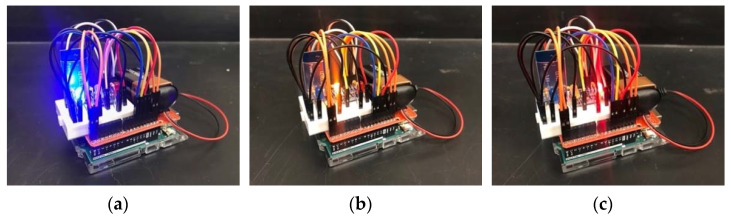
LED Subsystem with (**a**) Blue LED, (**b**) Yellow LED, and (**c**) Red LED.

Once we obtained the QFDC plots and their confusion matrices for each dataset, we selected the parameter set with the highest accuracy for the next and final step of the UAV failure detection system, where the LED subsystem uses three LEDs—blue LED, yellow LED, and red LED (see Figure 8).

The LED subsystem serves as a visual representation of the k-means clustering algorithm and establishes feasibility for the failure detection system. We wrote a different code in Arduino IDE for displaying the number of times each LED turns on (see Appendix A for the code). Boundary conditions specify lines that separate the three health state regions from the QFDC plot. We used the blue, yellow, and red LEDs for detecting the normal state, faulty state, and failure state, respectively. We conducted nine flights for one minute each, and took 100 samples for the three propeller cases with three trials each, yielding nine flights. Accuracies for the LED subsystem were determined based on the constraints for the LEDs.

## 5. Results

In this paper, the novel failure detection system uses an IMU sensor to measure in-flight vibration of the Parrot ANAFI quadcopter, detect failure using the k-means clustering algorithm, and reduce the number of crashes in faulty UAVs. After flying the Parrot ANAFI drone for fifteen flights, we obtained vibration graphs from the IMU sensor readings. We also obtained QFDC plots and confusion matrices using the k-means clustering algorithm based on the chosen data and parameter sets. Finally, we obtained results for the LED subsystem that tracks the number of LEDs that turn on.

### 5.1. Vibration Graphs

**Figure 9:**
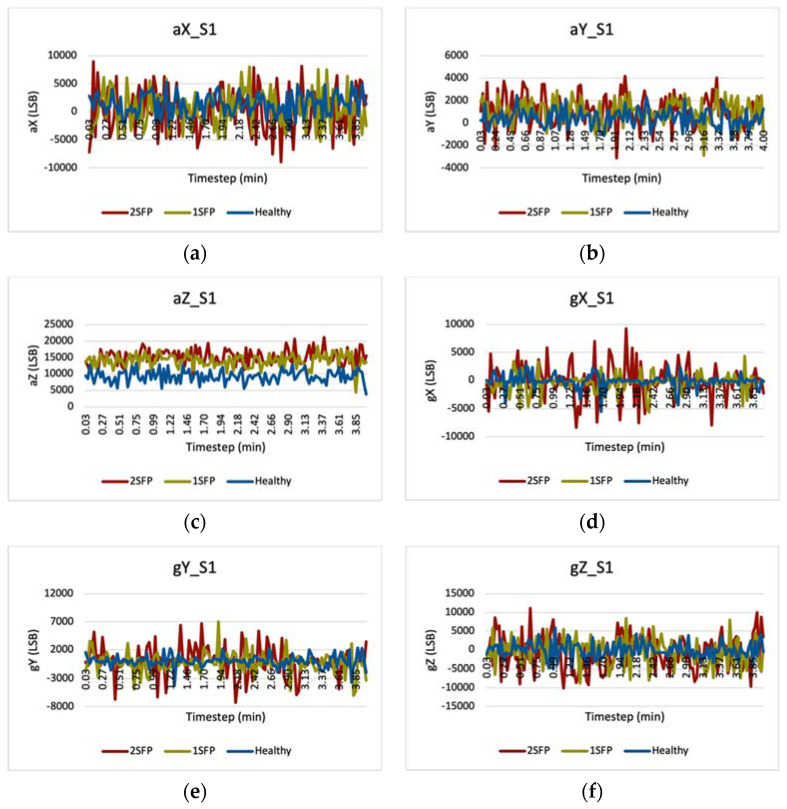
Measured Flight S1 Vibration Data for Parameter Sets (**a**) aX, (**b**) aY, (**c**) aZ, (**d**) gX, (**e**) gY, and (**f**) gZ.

Figure 9 shows the vibration graphs for a flight set that contain readings for acceleration and gyro in the *x*-, *y*-, and *z*-directions. We used the GY-521 sensor for measuring vibration data, and read these values in the Arduino IDE software. The independent variable is timestep in minutes, and the dependent variables are the six measured parameters with units in least significant bit (LSB). Each graph compares the vibration data for different propeller fault configurations—healthy (blue), 1SFP (yellow), and 2SFP (red). Note that we only show the vibration graphs for flight set S1, as all other flight sets (S2 to S5) show similar trends.

### 5.2. QFDC Plots

**Figure 10:**
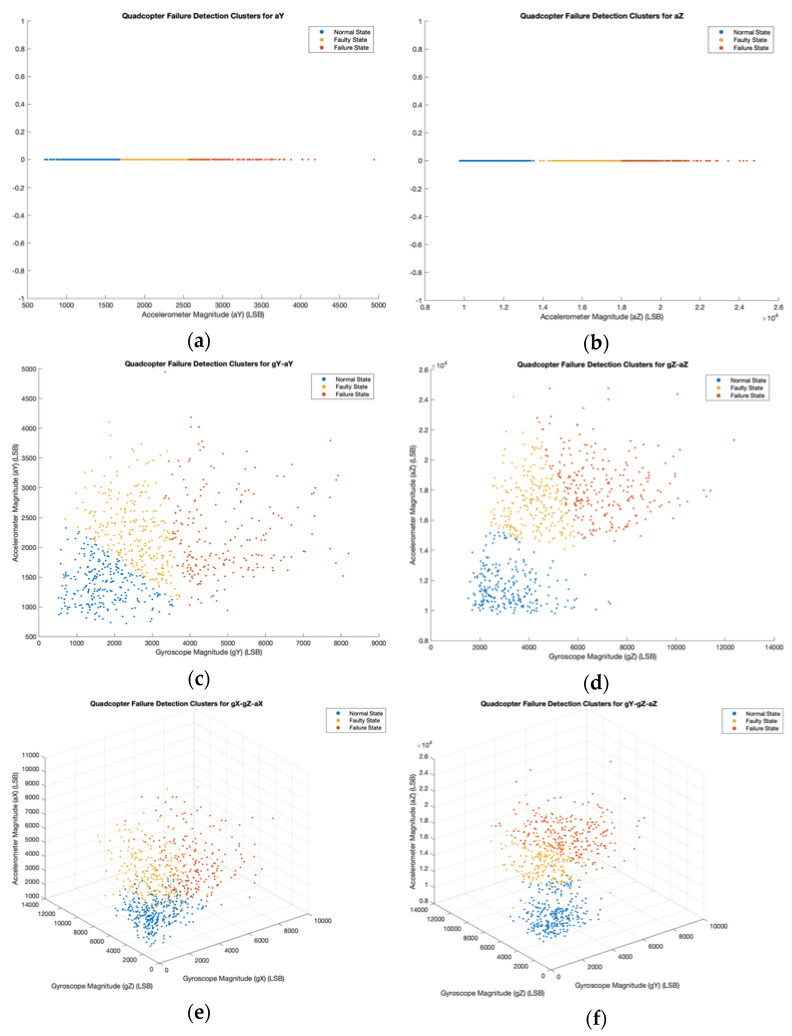
QFDC Plot for (**a**) aY, (**b**) aZ, (**c**) gY-aY, (**d**) gZ-aZ, (**e**) gX-gZ-aX, and (**f**) gY-gZ-aZ.

In Figure 10, we show the QFDC plots with three clusters (normal state, faulty state, and failure state) for the selected parameter sets from Table 2. Each dataset contained 750 data points, written as $\left(x_{1},\ldots ,x_{n}\right)$. We implemented the k-means clustering algorithm in MATLAB with *k* = 3 and *n* = 750, which allocated each data point into one of the three clusters. In Table 4, 3 × 3 confusion matrices for each QFDC plot compare the health states for each data point between the actual label and the clustering label. We obtain both QFDC plots and confusion matrices for *n* = 1, 2, and 3 dimensions, and we obtain only confusion matrices for *n* = 4, 5, and 6 dimensions.

Table 5 shows the performance metrics from Equations (5) to (8) for the nine parameter sets.

### 5.3. LED Subsystem

**Figure 11:**
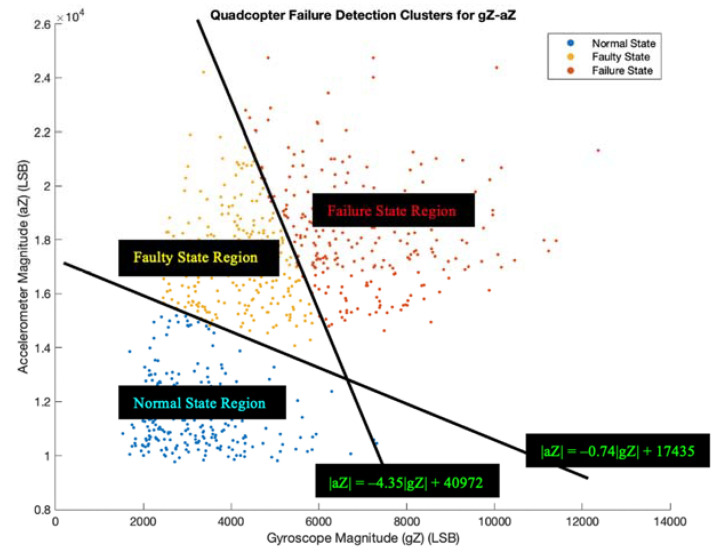
Chosen QFDC Plot with Boundary Conditions.

Since the parameter set gZ-aZ has the highest accuracy of 92.1% (see Table 5), we chose this parameter set to move forward with decision-making, the last step of the UAV failure detection system, using the LED subsystem. Three LEDs (blue, yellow, and red) turn on one at a time based on specified constraints. We set up boundary conditions with equations for two linear lines that separate the three different cluster regions: normal state region, faulty state region, and failure state region. Their equations are determined in slope-intercept form by using two points on a line that separate two regions, and we calculate their slope. Figure 11 shows the chosen QFDC plot with indicated regions and their linear equations.

The blue, yellow, and red LEDs correspond to normal state, faulty state, and failure state, respectively. We conducted nine more flights, where the Parrot drone flew based on the three propeller cases with three trials each. Flight F1 utilized the healthy propeller case, Flight F2 utilized the 1SFP case, and Flight F3 utilized the 2SFP case. The drone flew around for one minute, and we took 100 samples to track the number of LEDs that turned on during the flight (see Table 6). We calculated their accuracies by taking the quotient of the counted LED (bolded number) and the number of samples (100) (see Table 7). A video of the conducted flight experiments with LEDs is available in the Supplementary Materials section.

### 5.4. Discussion of Results

Figure 9 and Figure 10 showed vibration graphs, QFDC plots, and confusion matrices for the UAV failure detection system that utilized the k-means clustering algorithm as a part of the failure detection system to reduce the number of fatal UAV crashes. Graphs for the six parameters (aX, aY, aZ, gX, gY, and gZ) each compared the vibration of the Parrot quadcopter as a function of timestep. The measured vibration curves for the 2SFP case (red) have higher values than both the 1SFP (yellow) and healthy (blue) propeller cases. When the propeller blades exhibited damage, additional vibration signals resulted in these higher magnitudes and affected the aerodynamics of the quadcopter. The rotor(s) generated fluctuating thrust for the faulty propellers, which resulted in higher vibrations on the structure. In other words, faulty propellers distorted the quadcopter dynamics as it affected the thrust in the rotors, resulting in higher vibration. We considered different propeller fault configurations in UAV failure detection for conducting experimental flights; simulations were unnecessary because we could perform real flight experiments, thanks to the facility at Southern Illinois University Carbondale.

Each QFDC plot contained 750 points from datasets of selected parameters for dimensions between 1 and 6. Three health state clusters (normal state, faulty state, and failure state) contained these data points after running the k-means clustering algorithm. For *n* = 1, the QFDC plots were one-dimensional and arranged each data point in a horizontal line. For *n* = 2, *x*_1_ represented a gyroscope parameter, while *x*_2_ represented an accelerometer parameter. The QFDC plots showed these data points arranged in a two-dimensional plane. For *n* = 3, *x*_1_ and *x*_2_ represented the two gyroscope parameters, while *x*_3_ represented an accelerometer parameter. These plots showed data points scattered in a three-dimensional space. By looking at these QFDC plots, failure most likely occurred when each plot attained higher values for each chosen parameter. Each confusion matrix compared the health states for each data point between the actual label and the clustering label. For *n* = 4, 5, and 6, though we provided no QFDC plot for higher dimensions, their confusion matrices provided sufficient information for calculating performance metrics. The top three parameter sets are gZ-aZ, gY-gZ-aZ, and gY-gZ-aY-aZ, with accuracies of 92.1%, 81.7%, and 77.3%, respectively (Table 5).

In this study, we showed the acceleration and gyro parameter sets for each dimension, although other combinations are possible. In Table 5, the parameter sets that had parameter aZ attained higher accuracies. We considered gravity for measuring acceleration in the *z*-direction (aZ). Since vibration signals had no negative values, this parameter stands out among the other five that would make accuracies higher for these chosen parameter sets. As *n*: 1→2, the accuracy increases, but as *n*: 2→6, the accuracy decreases. One possible reason for *n* = 2 having the best performance metrics is the ability for the algorithm to better minimize the distance between data points and the centroid in MATLAB for this dimension. As shown in the confusion matrices in Table 4, maximizing the true positive (FF) and true negative (AA and NN) cells, and minimizing all other cells, would guarantee the highest accuracy possible for the UAV failure detection system. The parameter sets gY-aY and gZ-aZ had accuracies of 76.3% and 92.1%, respectively, for *n* = 2. Information for failure detection may become masked by unnecessary or redundant information for higher dimensions. Our investigations show that the parameter set gZ-aZ results in the highest failure detection accuracy. Hence, we selected that parameter set for the flight experiments.

We set boundary conditions in Figure 11 for the LED subsystem that confirmed the effectiveness of the UAV failure detection system. After conducting nine additional flight experiments, the accuracies ranged between 89% and 95%, (Table 7), and each of the nine accuracies were within the 3.5% difference of the gZ-aZ plot (92.1%). For Flight F1, the blue and yellow LEDs turned on, but not the red LED. As expected, no signs of failure occurred when the quadcopter blades were in good condition. For Flight F2, all three LEDs turned on, and possible failure occurred when the red LED blinked on at some point during the flight. In addition, the yellow and red LEDs turned on for Flight F3, which showed existing faults or failures from the damaged quadcopter blades. The written Arduino code correctly identified these health states. Our system detected failure in 0.6 s as we used only one sensor to measure the necessary parameters for the k-means clustering algorithm. By attaining these high accuracies for both the QFDC plot and the LED subsystem, the proposed UAV failure detection system shows its feasibility and effectiveness in detecting failures. We can use the outputs obtained from this failure detection system in the next step of the safety framework, which is finding a safe landing zone.

## 6. Conclusions

Safety concerns of flying UAVs in urban environments is addressed with a three-step safety framework that consists of (1) detecting failures, (2) finding an uncluttered emergency landing zone, and (3) navigating the UAV to the landing zone found in step 2. Here, to address the first step, we proposed a novel failure detection system for a UAV that detects propeller failures to reduce the risk of crashes and show its effectiveness in experimental flights. We built a hardware system consisting of an IMU sensor and a Bluetooth module, and connected them to the Arduino Uno for measuring the vibration signals of the quadcopter during the flights. We flew the quadcopter for three different propeller configurations based on the number of faulty propellers for a total of fifteen flights. Before reading the data, we measured the acceleration and gyro data in three directions with an IMU sensor and wirelessly sent the parameter data to Arduino IDE using Bluetooth. We extracted the data by selecting parameters and values for obtaining vibration graphs. The k-means clustering algorithm was successful in allocating data points in three clusters, representing different health states for each QFDC plot with different parameter combinations. We also used the LED subsystem with three LEDs as a visual representation of the algorithm for validating the failure detection system. We set boundary conditions from the corresponding QFDC plot and tracked each LED based on the written code during flight. After applying the k-means clustering algorithm, we discovered the best selection of parameters with high accuracy. We detected failure in 0.6 s, thus making the use of additional sensors unnecessary. By fulfilling the five steps of this system and using the k-means clustering algorithm, we achieved promising results for detecting UAV failure. Future scope of this work includes integrating this algorithm with the other two steps of the safety framework, where the researchers published the second step in ref. [8].

## Figures and Tables

**Table 1:** Comparison of Failure Detection Types with Related Works.

Reference	UAV	Type of Failure	Algorithm	Findings
Arasanipalai et al., 2020 [21]	Quadcopter	Propeller Failure	Recurrent Neural Network (RNN)	Propeller failure was detected in 2.5 s by using an RNN for two and three working propellers in a quadcopter
Bondyra et al., 2017 [25]	Quadcopter	Propeller Failure	Support Vector Machine (SVM)	The best performance label for failure detection is fast Fourier transform, even within 250 ms
Cheng et al., 2019 [4]	Quadcopter	Motor and Propeller Failure	Self-Organizing Map (SOM)	Based on the confusion matrix, the SOM model has an accuracy of 99%, and recall of failure situation is 100%
Dooraki et al., 2020 [22]	Quadcopter	Motor Failure	Fault-Tolerant Bio Inspired Flight Controller (FT-BFC)	FT-BFC maximizes the accumulated reward over time, and can reach the desired waypoint in a shorter time
Keipour et al., 2020 [19]	Fixed-wing	Control Surfaces Failure (Engine, Aileron, Rudder, Elevator)	No algorithm presented, only dataset provided	The failure ground-truth message happens within 0.2 s after the exact moment of the fault
Magsino et al., 2020 [32]	Octocopter	Motor Failure	Fuzzy Logic	The redundant flight recovery system is operational for *X*_odd_ and *X*_even_ flight attitudes, providing effective angle responses
Ray et al., 2021 [28]	Quadcopter	Motor Failure	Skewness Scanning	Parameters SA5 and SA6 for pitch ^1^ and SA5, SA6, and SA8 for yaw have precise skewness values and minimal errors for motor short turns
Zhang et al., 2021 [7]	Quadcopter	Propeller Failure	Long- and Short-Term Memory (LSTM) and Back Propagation (BP)	The LSTM model outperforms the BP model in time series classification, with accuracies of 96% and 65%, respectively

^1^ Skewness of Approximate Coefficients for Levels 5, 6, and 8 for pitch/yaw.

**Table 2:** Selection of Parameters and Dimensions.

Dimension	Parameter Set
1	aY
1	aZ
2	gY-aY
2	gZ-aZ
3	gX-gZ-aX
3	gY-gZ-aZ
4	gY-gZ-aY-aZ
5	gX-gY-gZ-aY-aZ
6	gX-gY-gZ-aX-aY-aZ

**Table 3:** General 3 × 3 Confusion Matrix.

	Clustering Label	Failure State	Faulty State	Normal State
Actual Label				
**Failure** **State**		FF	FA	FN
**Faulty** **State**		AF	AA	AN
**Normal** **State**		NF	NA	NN

**Table 4:** Confusion Matrices for (**a**) aY, (**b**) aZ, (**c**) gY-aY, (**d**) gZ-aZ, (**e**) gX-gZ-aX, (**f**) gY-gZ-aZ, (**g**) gY-gZ-aY-aZ, (**h**) gX-gY-gZ-aY-aZ, and (**i**) gX-gY-gZ-aX-aY-aZ.

	Clustering Prediction	Failure State	Faulty State	Normal State		Clustering Prediction	Failure State	Faulty State	Normal State
Actual Label					Actual Label				
**Failure** **State**		83	124	43	**Failure** **State**		141	109	0
**Faulty** **State**		53	120	77	**Faulty** **State**		53	197	0
**Normal** **State**		1	58	191	**Normal** **State**		0	12	238
(**a**)					(**b**)				
	**Clustering** **Prediction**	**Failure** **State**	**Faulty** **State**	**Normal** **State**		**Clustering** **Prediction**	**Failure** **State**	**Faulty** **State**	**Normal** **State**
**Actual** **Label**					**Actual** **Label**				
**Failure** **State**		170	80	0	**Failure** **State**		231	19	0
**Faulty** **State**		41	174	35	**Faulty** **State**		16	217	17
**Normal** **State**		0	22	228	**Normal** **State**		0	7	243
(**c**)					(**d**)				
	**Clustering** **Prediction**	**Failure** **State**	**Faulty** **State**	**Normal** **State**		**Clustering** **Prediction**	**Failure** **State**	**Faulty** **State**	**Normal** **State**
**Actual** **Label**					**Actual** **Label**				
**Failure** **State**		149	101	0	**Failure** **State**		201	49	0
**Faulty** **State**		49	129	72	**Faulty** **State**		37	171	42
**Normal** **State**		0	12	238	**Normal** **State**		0	9	241
(**e**)					(**f**)				
	**Clustering** **Prediction**	**Failure** **State**	**Faulty** **State**	**Normal** **State**		**Clustering** **Prediction**	**Failure** **State**	**Faulty** **State**	**Normal** **State**
**Actual** **Label**					**Actual** **Label**				
**Failure** **State**		172	78	0	**Failure** **State**		180	70	0
**Faulty** **State**		49	161	40	**Faulty** **State**		41	159	50
**Normal** **State**		0	3	247	**Normal** **State**		0	11	239
(**g**)					(**h**)				
	**Clustering** **Prediction**	**Failure** **State**	**Faulty** **State**	**Normal** **State**					
**Actual** **Label**									
**Failure** **State**		201	49	0					
**Faulty** **State**		37	171	42					
**Normal** **State**		0	9	241					
(**i**)									

**Table 5:** Performance Metrics for Parameter Sets.

*n*	Parameter Set	Recall	Precision	Accuracy	F-Score
1	aY	0.606	0.332	0.525	0.429
1	aZ	0.727	0.564	0.768	0.635
2	gY-aY	0.806	0.680	0.763	0.738
2	gZ-aZ	0.935	0.924	0.921	0.930
3	gX-gZ-aX	0.753	0.596	0.688	0.665
3	gY-gZ-aZ	0.844	0.804	0.817	0.824
4	gY-gZ-aY-aZ	0.778	0.688	0.773	0.730
5	gX-gY-gZ-aY-aZ	0.814	0.720	0.771	0.764
6	gX-gY-gZ-aX-aY-aZ	0.766	0.680	0.757	0.720

**Table 6:** Tracking Three LEDs from Nine Flights.

Flight		Blue LED	Yellow LED	Red LED
F1	Trial 1	95	5	0
	Trial 2	94	6	0
	Trial 3	94	6	0
F2	Trial 1	2	94	4
	Trial 2	4	92	4
	Trial 3	3	93	4
F3	Trial 1	0	8	92
	Trial 2	0	7	89
	Trial 3	0	11	93

**Table 7:** LED Subsystem Accuracies.

Flight	Trial 1	Trial 2	Trial 3
F1	0.95	0.94	0.94
F2	0.94	0.92	0.93
F3	0.92	0.89	0.93

## Supplementary Materials

The following supporting information can be downloaded at: youtube.com/watch?v=jMxcHfU0l-s, The video shows flight experiments with LEDs to confirm the feasibility of using the proposed method in detecting failures in UAVs.

## Appendix A

Developed code for LED Subsystem

#include “Wire.h” const int MPU_ADDR = 0x68; int16_t accelerometer_x, accelerometer_y, accelerometer_z; int16_t gyro_x, gyro_y, gyro_z; char tmp_str[7]; char* convert_int16_to_str(int16_t i) { sprint(tmp_str, “%6d”, i); return tmp_str; } int i; int LED1 = 11; //Blue LED from Pin 11 for Normal Condition int LED2 = 12; //Yellow LED from Pin 12 for Faulty Condition int LED3 = 13; //Red LED from Pin 13 for Failure Condition int BlueLEDon = 0; int YellowLEDon = 0; int RedLEDon = 0; void setup() { Serial.begin(9600); Wire.begin(); Wire.beginTransmission(MPU_ADDR); Wire.write(0x6B); Wire.write(0); Wire.endTransmission(true); pinMode(LED1, OUTPUT); pinMode(LED2, OUTPUT); pinMode(LED3, OUTPUT); for(i = 1; i < 101; i++) //Read 100 lines once for each flight { Wire.beginTransmission(MPU_ADDR); Wire.write(0x3B); Wire.endTransmission(false); Wire.requestFrom(MPU_ADDR, 7*2, true); accelerometer_x = Wire.read()<<8 | Wire.read(); accelerometer_y = Wire.read()<<8 | Wire.read(); accelerometer_z = Wire.read()<<8 | Wire.read(); gyro_x = Wire.read()<<8 | Wire.read(); gyro_y = Wire.read()<<8 | Wire.read(); gyro_z = Wire.read()<<8 | Wire.read(); delay(600); Serial.print(i); Serial.print(“ | aZ = “); Serial.print(convert_int16_to_str(accelerometer_z)); Serial.print(“ | gZ = “); Serial.print(convert_int16_to_str(gyro_z)); if(abs(accelerometer_z) < - –0.74*abs(gyro_z) + 17435) //Boundary Condition #1 { digitalWrite(LED1, HIGH); //Turn Blue LED on digitalWrite(LED2, LOW); //Turn Yellow LED off digitalWrite(LED3, LOW); //Turn Red LED off BlueLEDon = BlueLEDon + 1; } else { if(abs(accelerometer_z) < –4.35*abs(gyro_z) + 40972) //Boundary Condition #2 { digitalWrite(LED1, LOW); //Turn Blue LED off digitalWrite(LED2, HIGH); //Turn Yellow LED on digitalWrite(LED3, LOW); //Turn Blue LED off YellowLEDon = YellowLEDon + 1; } else //Boundary Condition #3 { digitalWrite(LED1, LOW); //Turn Blue LED off digitalWrite(LED2, LOW); //Turn Yellow LED off digitalWrite(LED3, LOW); //Turn Red LED on RedLEDon = RedLEDon + 1; } } Serial.print(“ | Blue LED: “); Serial.print(BlueLEDon); Serial.print(“ | Yellow LED: “); Serial.print(YellowLEDon); Serial.print(“ | Red LED: “); Serial.print(RedLEDon); Serial.println(); } }

## References

[1] Kandaswamy G., Balamuralidhar P. (2017). Health Monitoring and Failure Detection of Electronic and Structural Components in Small Unmanned Aerial Vehicles. Int. J. Mech. Mechatron. Eng..

[2] Bektash O., Cour-Harbo A.l. Vibration Analysis for Anomaly Detection in Unmanned Aircraft. Proceedings of the Annual Conference of the Prognostics and Health Management Society.

[3] Bektash O., Pedersen J.N., Gomez A.R., Cour-Harbo A.l. Automated Emergency Landing System for Drones: SafeEYE Project. Proceedings of the 2020 International Conference on Unmanned Aircraft Systems (ICUAS).

[4] Cheng D.L., Lai W.H. Application of Self-Organizing Map on Flight Data Analysis for Quadcopter Health Diagnosis System. International Archives of the Photogrammetry, Remote Sensing & Spatial Information Sciences, Proceedings of the International Society of Photogrammetry and Remote Sensing (ISPRS), Enschede, The Netherlands, 10–14 June 2019.

[5] Qi J., Song D., Wu C., Han J., Wang T. (2012). KF-Based Adaptive UKF Algorithm and its Application for Rotorcraft UAV Actuator Failure Estimation. Int. J. Adv. Robot. Syst..

[6] Rago C., Prasanth R., Mehra R., Fortenbaugh R. Failure Detection and Identification and Fault Tolerant Control using the IMM_KF with Applications to the Eagle-Eye UAV. Proceedings of the 37th IEEE Conference on Decision & Control.

[7] Zhang X., Zhao Z., Wang Z., Wang X. (2021). Fault Detection and Identification Method for Quadcopter Based on Airframe Vibration Signals. Sensors.

[8] Nepal U., Eslamiat H. (2022). Comparing YOLOv3, YOLOv4 and YOLOv5 for Autonomous Landing Spot Detection in Faulty UAVs. Sensors.

[9] Qi X., Theilliol D., Qi J., Zhang Y., Han J., Song D. Fault Diagnosis and Fault Tolerant Control Methods for Manned and Unmanned Helicopters: A Literature Review. Proceedings of the 2013 Conference on Control and Fault-Tolerant Systems (SysTol).

[10] Saied M., Lussier B., Fantoni I., Shraim H., Francis C. (2017). Fault Diagnosis and Fault-Tolerant Control of an Octorotor UAV using Motors Speeds Measurements. IFAC Pap..

[11] Ghalamchi B., Jia Z., Mueller M.W. (2020). Real-Time Vibration-Based Propeller Fault Diagnosis for Multicopters. IEEE/ASME Trans. Mechatron..

[12] Nguyen N.P., Hong S.K. (2018). Sliding Mode Thau Observer for Actuator Fault Diagnosis of Quadcopter UAVs. Appl. Sci..

[13] Avram R.C., Zhang X., Campbell J., Muse J. (2015). IMU Sensor Fault Diagnosis and Estimation for Quadrotor UAVs. IFAC Pap..

[14] Zermani S., Dezan C., Euler R. Embedded Decision Making for UAV Missions. Proceedings of the 2017 6th Mediterranean Conference on Embedded Computing (MECO).

[15] Sujit P.B., Sousa J.B. Multi-UAV Task Allocation with Communication Faults. Proceedings of the 2012 American Control Conference (ACC).

[16] Boroujeni S., Etemad S.A., Whitehead A. Robust Horizon Detection using Segmentation for UAV Applications. Proceedings of the 2012 Ninth Conference on Computer and Robot Vision.

[17] Fan H., Fang H., Dong Y., Shi H., Ren S. UAV Engine Fault and Diagnosis with Parameter Models based on Telemetry Data. Proceedings of the 2017 Prognostics and System Health Management Conference (PHM-Harbin).

[18] Zhao Z., Zhou R., Dong Z. Aero-Engine Faults Diagnosis Based on K-Means Improved Wasserstein GAN and Relevant Vector Machine. Proceedings of the 2019 Chinese Control Conference (CCC).

[19] Keipour A., Mousaei M., Scherer S. (2020). ALFA: A Dataset for UAV Fault and Anomaly Detection. Int. J. Robot. Res..

[20] Lin C.E., Shao P.C. (2020). Failure Analysis for an Unmanned Aerial Vehicle using Safe Path Planning. J. Aerosp. Inf. Syst..

[21] Arasanipalai R., Agrawal A., Ghose D. (2020). Mid-flight Propeller Failure Detection and Control of Propeller-Deficient Quadcopter using Reinforcement Learning. arXiv.

[22] Dooraki A.R., Lee D.J. Reinforcement Learning Based Flight Controller Capable of Controlling a Quadcopter with Four, Three, and Two Working Motors. Proceedings of the 2020 20th International Conference on Control, Automation and Systems (ICCAS).

[23] Iannace G., Ciaburro G., Trematerra A. (2019). Fault Diagnosis for UAV Blades using Artificial Neural Network. Robotics.

[24] Ghalamchi B., Mueller M. Vibration-Based Propeller Fault Diagnosis for Multicopters. Proceedings of the 2018 International Conference on Unmanned Aircraft Systems (ICUAS).

[25] Bondyra A., Gasior P., Gardecki S., Kasinki A. Fault Diagnosis and Condition Monitoring of UAV Rotor using Signal Processing. Proceedings of the 2017 Signal Processing: Algorithms, Architectures, Arrangements, and Applications (SPA).

[26] Banerjee P., Okolo W., Moore A. (2020). In-Flight Detection of Vibration Anomalies in Unmanned Aerial Vehicles. J. Nondestruct. Eval. Diagn. Progn. Eng. Syst..

[27] Du C., Zhang X., Zhong R., Li F., Yu F., Rong Y., Gong Y. (2022). Unmanned Aerial Vehicle Rotor Fault Diagnosis Based on Interval Sampling Reconstruction of Vibration Signals and a One-Dimensional Convolutional Neural Network Deep Learning Method. Meas. Sci. Technol..

[28] Ray D.K., Roy T., Chattopadhyay S. (2021). Skewness Scanning for Diagnosis of a Small Inter-Turn Fault in Quadcopter’s Motor Based on Motor Current Signature Analysis. IEEE Sens. J..

[29] Souza J.S., Bezerril M.C., Silva M.A., Veras F.C., Lima-Filho A., Ramos J.G., Brito A.V. (2021). Motor Speed Estimation and Failure Detection of Small UAV using Density of Maxima. Front. Inf. Technol. Electron. Eng..

[30] Veras F.C., Lima T.L.V., Souza J.S., Ramos J.G.G.S., Filho A.C.L., Brito A.V. (2019). Eccentricity Failure Detection of Brushless DC Motors From Sound Signals Based on Density of Maxima. IEEE Access.

[31] Ambroziak L., Simha A., Pawluszewicz E., Kotta Ü., Bożko A., Kondratiuk M. Motor Failure Tolerant Control System With Self Diagnostics for Unmanned Multirotors. Proceedings of the 2019 24th International Conference on Methods and Models in Automation and Robotics (MMAR).

[32] Magsino E.R., Say M.F., Tan J.A. Achieving Complete UAV Delivery in the Presence of Motor Failures. Proceedings of the 2020 IEEE 10th Symposium on Computer Applications & Industrial Electronics (ISCAIE).

[33] Hu Z., Zhu J., Wang H. Experimental Research on UAV Stability based on Vibration Signal Measurement of UAV Motors. Proceedings of the 2021 3rd International Symposium on Robotics & Intelligent Manufacturing Technology (ISRIMT).

[34] Pourpanah F., Zhang B., Ma R., Hao Q. Anomaly Detection and Condition Monitoring of UAV Motors and Propellers. Proceedings of the 2018 IEEE Sensors.

[35] Na S., Xumin L., Yong G. Research on k-means Clustering Algorithm: An Improved k-means Clustering Algoritm. Proceedings of the 2010 Third International Symposium on Intelligent Information Technology and Security Informatics.

